# Structural Refinement of Carbimazole by NMR Crystallography

**DOI:** 10.3390/molecules26154577

**Published:** 2021-07-29

**Authors:** Andrea Scarperi, Giovanni Barcaro, Aleksandra Pajzderska, Francesca Martini, Elisa Carignani, Marco Geppi

**Affiliations:** 1 Department of Chemistry and Industrial Chemistry, University of Pisa, Via G. Moruzzi 13, 56124 Pisa, Italy; a.scarperi@studenti.unipi.it (A.S.); francesca.martini@unipi.it (F.M.); 2 Institute For Chemical And Physical Processes, Italian National Council for Research, CNR/IPCF, Via G. Moruzzi 1, 56124 Pisa, Italy; barcaro@ipcf.cnr.it; 3 Department of Radiospectroscopy, Faculty of Physics, Adam Mickiewicz University, Uniwersytetu Poznanskiego 2, 61-614 Poznan, Poland; aleksandra.pajzderska@amu.edu.pl; 4 Center for Instrument Sharing, University of Pisa (CISUP), 56126 Pisa, Italy; 5 Institute for the Chemistry of OrganoMetallic Compounds, Italian National Council for Research, CNR/ICCOM, Via G. Moruzzi 1, 56124 Pisa, Italy

**Keywords:** crystalline drugs, pharmaceuticals, structure optimization, solid state NMR, CP-MAS, ^1^H-^13^C 2D-HETCOR, ^1^H CRAMPS, ^1^H-^1^H DQSQ, DFT calculations, isotropic chemical shift

## Abstract

The characterization of the three-dimensional structure of solids is of major importance, especially in the pharmaceutical field. In the present work, NMR crystallography methods are applied with the aim to refine the crystal structure of carbimazole, an active pharmaceutical ingredient used for the treatment of hyperthyroidism and Grave’s disease. Starting from previously reported X-ray diffraction data, two refined structures were obtained by geometry optimization methods. Experimental ^1^H and ^13^C isotropic chemical shift measured by the suitable ^1^H and ^13^C high-resolution solid state NMR techniques were compared with DFT-GIPAW calculated values, allowing the quality of the obtained structure to be experimentally checked. The refined structure was further validated through the analysis of ^1^H-^1^H and ^1^H-^13^C 2D NMR correlation experiments. The final structure differs from that previously obtained from X-ray diffraction data mostly for the position of hydrogen atoms.

## 1. Introduction

In the determination of the solid-state structure of crystalline compounds, NMR crystallography [1] has gradually grown in importance and is now considered complementary and supplementary to X-ray diffraction crystallography, the established leading technique in the field. The combination of the two techniques is particularly powerful in providing in-depth analyses of crystalline materials. Indeed, NMR techniques can cope with some limitations of X-ray diffractometry (XRD), such as the requirement of high quality and large single crystals. Of course, powder X-ray diffraction (PXRD) can also be applied in this case, but solving structure from PXRD still remains a challenging operation and the obtained structures are usually of lower quality than those derived from single crystal diffraction data. More importantly, XRD can have difficulty in making distinctions between isoelectronic species and atoms with similar atomic numbers. Finally, hydrogen atoms are poorly localized by XRD, as diffractometric techniques locate the centroid of the electron density, not the nuclear positions. On the other hand, NMR is intrinsically sensitive to the nuclear species and provides chemically selective information. In particular, the positions and interactions of hydrogen atoms can be finely probed by ^1^H NMR, and also exploiting heteronuclei, such as ^13^C, ^14/15^N, ^31^P and others.

In some research fields, the accurate characterization of the three-dimensional structure of solids is extremely important. This is particularly true in the pharmaceutical field, where the presence of an unknown polymorph could lead to extremely serious consequences [2,3]. Moreover, accurate crystal structures of solid pharmaceutics can be used to calculate important parameters using periodic density functional theory (DFT) calculations [4,5,6]. To this aim, structural studies capable of obtaining additional information to those derived from only diffractographic techniques are often required. 

NMR has provided crystallographic information from its earliest days [7]; nowadays, thanks to the enormous developments made in the NMR field, there are many examples in the literature where NMR crystallography was successfully applied in the field of biochemistry [8,9], in the study of inorganic materials [10], crystalline microporous materials [11], supramolecular assemblies [12], and pharmaceutical systems [13,14].

The ability to calculate NMR parameters, thanks to the improved efficiency of DFT codes, and in particular, thanks to the development of the gauge-including projector-augmented waves (GIPAW) method, has allowed rapid development of NMR crystallography, especially for the study of small organic molecules [15]. With respect to methods based on gauge-including atomic orbitals (GIAOs) [16], although extended to account for periodic conditions [17], in recent years, GIPAW has become the most popular method, as a first-principle theoretical framework in the context of NMR crystallography [18,19]. The reason for its success stems from the development of well-developed codes using periodic boundary conditions in conjunction with plane-waves, as basis sets and accurate dedicated pseudopotentials to build Bloch states, and simulate magnetic properties of crystalline (and, more in general, solid-state) systems. In this area, NMR methods have been used in a wide range of applications, for example assisting the structure solution process from PXRD data [20,21,22], establishing molecular conformations [23,24], confirming and rationalizing intermolecular interactions [25,26,27], and in some cases, deriving complete structures in absence of diffraction data [28,29,30].

In this context, an important research field regards the validation of structures derived from diffractographic data. This process often results in the optimization of atom positions in the unit cell of the crystal. The validation of diffractographic structures is based on the comparison between experimentally measured NMR parameters with those calculated with DFT methods. This process was proven effective at resolving ambiguities related to the molecular structure [31], to choose between alternative proposed structures [32,33], and to refine them through optimization of atom positions in the unit cell [34,35,36,37]. Although optimization of hydrogen atoms usually has the biggest effect, changes in heavy atom positions obtained through full optimization of the molecule sometimes results in improved agreement with experimental NMR data.

The present article reports the structural refinement of carbimazole by NMR crystallography. Carbimazole is, currently, one of the most used drugs for the treatment of hyperthyroidism and Grave’s disease. Its antithyroid action is attributed to its metabolization to methimazole in the body, which inhibits the first step of thyroid hormone synthesis in thyroglobulin [38,39,40,41]. Although anti-thyroid drugs (methimazole, carbimazole, propylthiouracil) have been used for over 70 years, despite a lot of research, their mechanisms of action are still not fully understood, especially at the molecular level.

The crystal structure of carbimazole has been independently studied by two research groups [42,43], and is reported in the Cambridge Structural Database (JOVDIH and JOVDIH01). The two structures are very similar and their main parameters are reported in Table 1. Delage et al. [42] derived the crystal structure by single crystal XRD (SCXRD) at an ambient temperature with CuKα irradiation in 1990, while the structural determination performed by D. Das and co-workers [43] with X-ray diffraction was a secondary aspect of a more general study, looking at the biological activity of carbimazole and its analogues. Therefore, the diffractometric procedure and the determined structure were not described and discussed in detail. In addition, to the best of our knowledge, no solid state NMR spectra of carbimazole are present in the literature thus far. All of the mentioned aspects make carbimazole an interesting case of study for structural refinement by NMR crystallography.

Here we present the first solid-state NMR (SSNMR) characterization of carbimazole. In particular, ^13^C cross polarization (CP)/magic angle spinning (MAS), ^1^H MAS, ^1^H combined rotation and multiple pulse spectroscopy (CRAMPS), ^1^H-^1^H double quantum-single quantum (DQSQ), and ^1^H-^13^C heteronuclear correlation (HETCOR) experiments were performed, and a complete assignment of the NMR peaks was achieved. The structural refinement was performed by using DFT with PAW pseudopotentials by optimizing hydrogen atoms only or all atoms in the cell. ^1^H and ^13^C isotropic chemical shifts were calculated for the raw and refined structures. The comparison between experimentally measured and calculated chemical shift values confirmed the better quality of the refined structures. These were further validated through the analysis of 2D NMR correlation ^1^H-^1^H DQSQ and ^1^H-^13^C HETCOR experiments.

## 2. Results

### 2.1. DSC, TGA and PXRD

**Figure 1:**
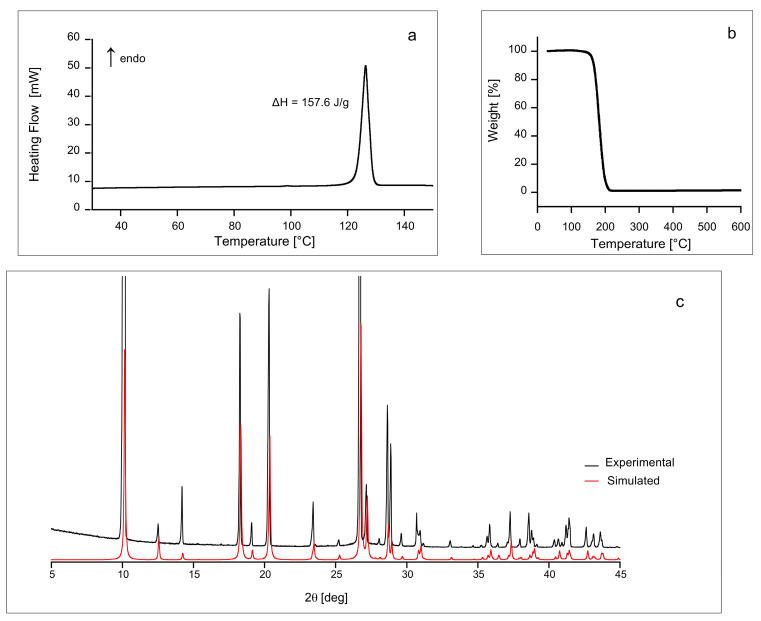
DSC thermogram (**a**), TGA (**b**), and experimental and simulated PXRD spectra (**c**) of carbimazole.

First, we performed differential scanning calorimetry (DSC), thermogravimetric analysis (TGA) and PXRD in order to obtain a basic characterization of our carbimazole sample. All of these experiments indicate that the sample under study is a pure, crystalline, and anhydrous form. In particular, the DSC thermogram (Figure 1a) shows the melting peak of carbimazole at 126.4 °C, in agreement with the value reported in DrugBank [44], equal to 123.5 °C. In addition, DSC and TGA (Figure 1b) do not show anomalies or weight loss around 100 °C or below, confirming that the investigated sample is anhydrous. PXRD spectrum also confirms that the solid form investigated is the same polymorph studied by Delage et al. [42] and Das et al. [43].

### 2.2. 1D High-Resolution ^13^C and ^1^H SSNMR Spectra

**Figure 2:**
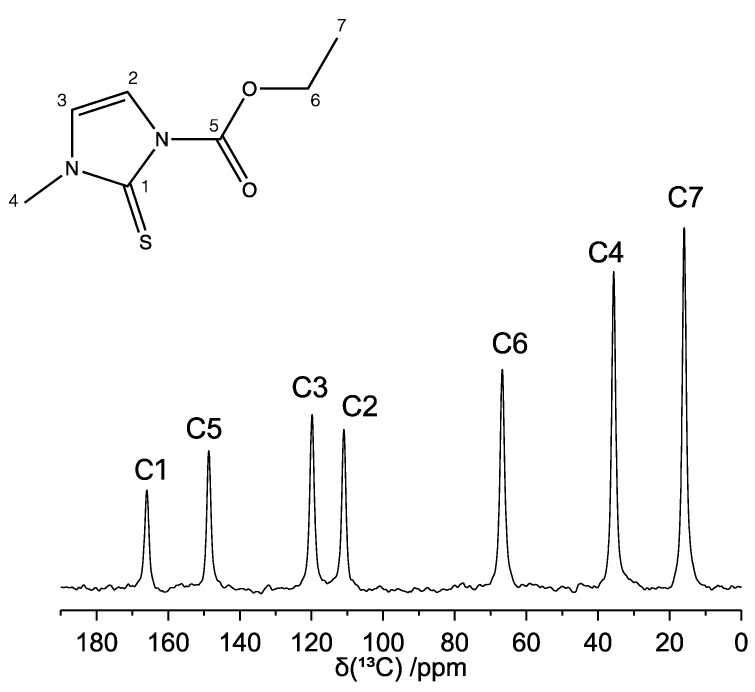
^13^C CP-MAS NMR spectrum of carbimazole (ν_MAS_ = 22 kHz). The assignment of the peaks is reported on the spectrum and refers to the labeling of the atoms as indicated in the chemical structure.

The ^13^C CP-MAS spectrum of carbimazole recorded at room temperature and at a MAS frequency (ν_MAS_) of 22 kHz is reported in Figure 2, together with the signals assignment, and shows seven narrow and well-resolved peaks. The absence of multiplicity of resonance of the signals confirms the presence of a single independent molecule in the unit cell (Z′ = 1), as previously reported by Delage and co-workers [42]. The assignment of the spectrum was carried out by comparison with the ^13^C solution-state NMR spectrum of carbimazole [43,45] and was confirmed by the ^1^H-^13^C HETCOR experiment (vide infra). It is worth noting that the signal intensities in the ^13^C CP-MAS spectrum reflect the number of protons directly linked to the carbon nuclei, as expected.

**Figure 3:**
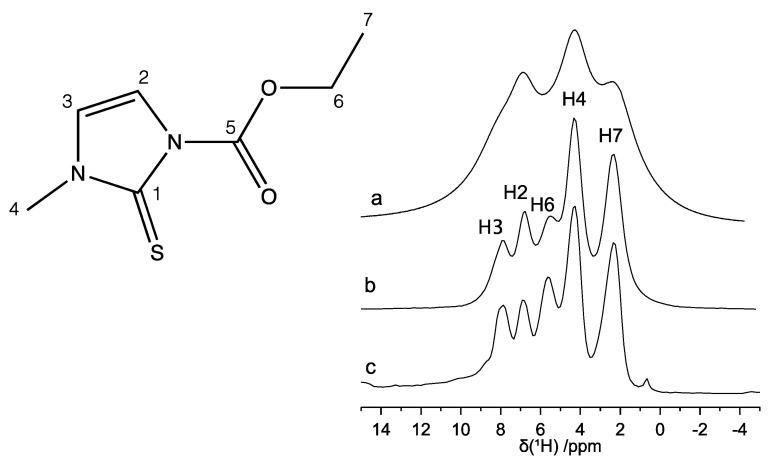
^1^H NMR spectra of carbimazole: (**a**) MAS (ν_MAS_ = 22 kHz), (**b**) PMLG-MAS (ν_MAS_ = 15 kHz), and (**c**) DUMBO-MAS (ν_MAS_ = 12 kHz). The assignment of the peaks is reported on the spectra and refers to the labeling of the atoms indicated in the chemical structure.

The ^1^H MAS spectrum recorded at ν_MAS_ = 22 kHz (Figure 3a) shows a scarce resolution; nevertheless, at least three heavily superimposed peaks centered at 2.4, 4.3, and 6.9 ppm can be identified. In order to improve the spectral resolution, MAS had to be combined with suitable pulse sequences, such as the phase modulated Lee-Goldburg (PMLG) and decoupling using mind boggling optimization (DUMBO), aimed at better removing the ^1^H homonuclear dipolar coupling. The spectra so obtained are reported in Figure 3b,c, respectively. Both PMLG-MAS and DUMBO-MAS spectra show greatly improved spectral resolution: five partially overlapped peaks are now clearly distinguishable, corresponding to the five groups of inequivalent protons, as expected on the basis of the molecular structure. As for the ^13^C spectrum, also in this case, the spectral assignment was performed by comparison with the ^1^H solution-state NMR spectra [43,46] and with the assistance of the ^1^H-^13^C HETCOR experiment. All experimental isotropic ^1^H and ^13^C chemical shift values are reported in Table 2 along with the assignment of the peaks.

### 2.3. Optimization of the Crystallographic Structure

Two crystal structures of carbimazole exist in the literature (JOVDIH [42] and JOVDIH01 [43]). They are in fair agreement on the values of the length of the axes of the orthorhombic unit cell belonging to the Pmmm point group (space groups Pna21 and Pnma for JOVDIH and JOVDIH01, respectively), measuring 7.689 Å, 6.637 Å, and 17.364 Å for JOVDIH and 7.698 Å, 6.650 Å, and 17.388 Å for JOVDIH01. The unit cell contains four molecules (for a total of 88 atoms) generated from a single independent (Z′ = 1) molecule via the symmetry operations of the point group (see Table 1). We focused our analysis on the estimation of the isotropic chemical shifts of both the ^13^C and ^1^H nuclei, which are reported in Table 2, by starting from the more recent JOVDIH01 crystal structure. All of the simulations were performed by imposing the experimental values of the length of the crystal axes. Within the cell, we used three levels of local optimization: (i) no optimization at all, by considering the experimental positions derived directly from the X-ray structure; (ii) a local optimization of the H atoms only (X-ray experimental C-H distances are in fact underestimated by about 10% with respect to known typical values); (iii) a complete local optimization of all the atoms within the unit cell. The data collected from the DFT-GIPAW simulation were corrected by using linear regression functions, whose analytic expressions are reported for each case in Table 5. Since the validation of the refined structures is mainly based on the values of RMSD between calculated shielding values and experimental chemical shifts, the approach to perform a separate regression for each set of data allowed systematic deviations to be minimized. As can be immediately evinced from the reported values, simulated isotropic chemical shifts estimated on the bare experimental positions carry a significant RMSD for both ^1^H and ^13^C species, due to the aforementioned underestimation of the C-H distances; moreover, the angular coefficient of the regression function results remarkably far from unity for both species, indicating the difficulty of reconciling experimental and simulated values in this case. The situation is improved when considering level (ii) and (iii) of local optimization: RMSDs are reduced to the typical values reported in the literature for the two considered species [47,48], and the analytical regression functions are characterized by angular coefficients near unity. Interestingly, the lowest RMSD value is achieved when only H atoms are optimized, indicating that the optimization of the heavier atoms at the DFT-GGA level slightly worsen the agreement with the experimental values. This can be expected as hybrid XC-functionals are more accurate in predicting chemical shifts for this type of molecule [49], but the chosen periodic approach allowed the use of the gradient-corrected functional only. PXRD patterns were also simulated for the three levels of optimization and they resulted in being very similar to each other (see Figure 1 and Supplementary Materials).

### 2.4. 2D SSNMR Spectra: Validation of the Optimized Structure

^1^H-^13^C HETCOR and ^1^H-^1^H DQSQ experiments are often applied to enlarge the amount of information in NMR crystallography studies [50,51,52]. Here, these experiments were performed in order to obtain a validation of the refined structure of carbimazole by a semi-quantitative comparison of the signal intensities in the spectra with the distances of the corresponding coupled nuclei measured from the optimized crystal structure. 

**Figure 4:**
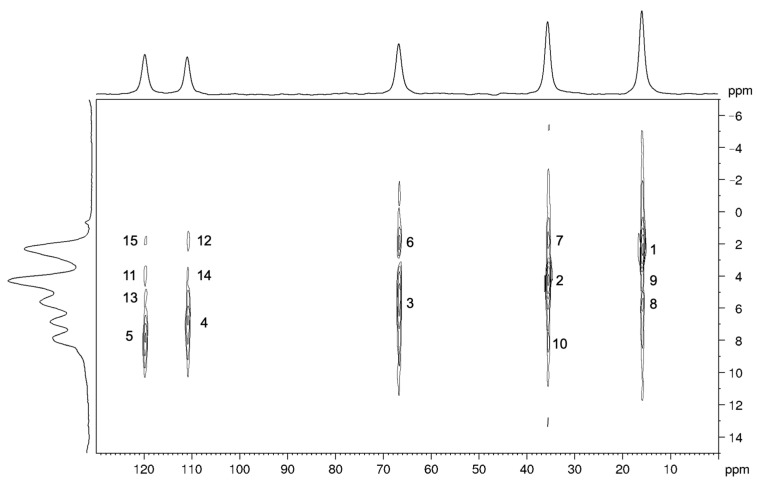
^1^H-^13^C MAS NMR HETCOR spectrum of carbimazole recorded at a spinning frequency of 15 kHz. The signals are numbered in order of decreasing intensity.

**Figure 5:**
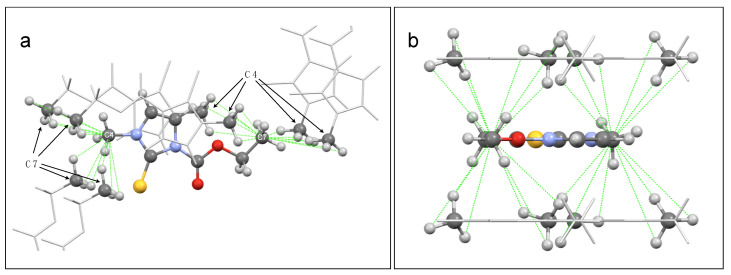
Two different views (**a**,**b**) of the 3D refined structure of carbimazole: a central molecule is highlighted (balls and sticks), together with the methyl groups of surrounding molecules, and C7-H4 and C4-H7 intermolecular distances are indicated (green dotted lines).

The ^1^H-^13^C HETCOR spectrum (Figure 4) shows signals whose intensities depend on the strength of the heteronuclear dipolar interactions, and, in turn, primarily depend on the distance of the coupled nuclei (Table 3). Indeed, the most intense peaks correspond to the directly bonded pairs of ^1^H-^13^C nuclei (peaks 1, 2, 3, 4, and 5). Among these peaks, the intensity is roughly proportional to the number of hydrogen atoms directly bonded to the carbon nucleus: maximum for the signals of the methyl groups and minimum for those of the olefinic groups. Among the other peaks, the signals with larger intensities are those corresponding to the intramolecular interactions C6-H7 and C7-H6 (peaks 6 and 8, respectively) and to the intermolecular interactions C4-H7* and C7-H4* (peaks 7 and 9, respectively). The C4-H7*/C7-H4* pairs show larger signal intensities than other pairs of nuclei characterized by shorter internuclear distances, since, in the crystal structure, there are 12 H4 atoms at distances shorter than 5 Å from each C7 (and vice versa there are 12 H7 atoms at distances shorter than 5 Å from each C4, as shown in Figure 5). The remaining signals in the spectrum also show intensities compatible with the C-H distances obtained from the optimized crystal structure. 

**Figure 6:**
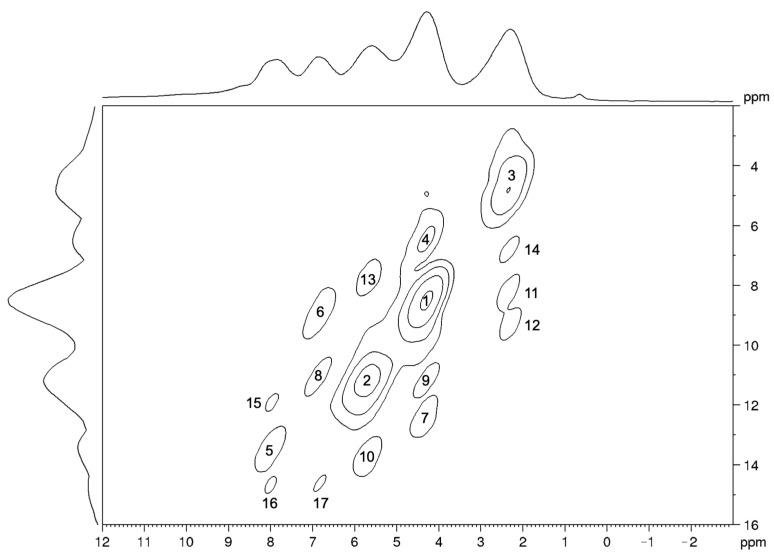
^1^H-^1^H MAS NMR DQSQ spectrum of carbimazole (ν_MAS_ = 12 kHz). The signals are numbered in order of decreasing intensity. The base contour level corresponds to 16% of the maximum spectral intensity; the multiplying factor for level increment is 1.8.

In the ^1^H-^1^H DQSQ spectrum (Figure 6), the signals corresponding to the interactions between ^1^H nuclei belonging to the same chemical group (peaks 1, 2, and 3) are characterized by the largest intensities, since the distances between these pairs of nuclei are smaller than any other. The difference in intensity between peaks 1 and 3, both arising from a methyl group, clearly suggests interpreting the intensity of the signals only in a semi-quantitative manner. Contrary to the HETCOR experiment, in fact, in the DQSQ spectrum, a correspondence between the trends of the minimum distances of H pairs and of the intensities of the corresponding signals in the spectrum cannot be established (Table 4). In any case, only the signals arising from ^1^H pairs showing a distance smaller than 2.9 Å in the optimized structure can be clearly detected in the DQSQ spectrum. Even in this case, the strongest intermolecular dipolar interactions are those between the protons belonging to the methyl groups 4 and 7 (Figure 5).

Although we could not interpret the signal intensities of the 2D spectra in a strictly quantitative way, these experiments were useful, on the one hand, to support the signal assignment of the ^1^H and ^13^C 1D high-resolution spectra, and on the other hand, to validate the crystal structure optimized by DFT. The observed deviations from the theoretical relationship between signal intensity and the inverse of the third power of the internuclear distance can have different sources. First, due to the difficulty of resolving all peak superpositions in the 2D spectra, the intensities were taken as heights of the unresolved 2D peaks. Second, the distances are calculated from an ideal “frozen” structure, so the real presence, at the experimental room temperature, of both rotational and vibrational molecular motions, certainly introduces discrepancies between actual and calculated internuclear distances. Although such discrepancies could be, in principle, strongly reduced by combining ab initio Molecular Dynamics within DFT GIPAW calculations, this is computationally very demanding and, in any case, beyond the scope of this work [53]. Third, possible artifacts can arise from the experiments, due, for instance, to RF inhomogeneity.

## 3. Materials and Methods

**Sample.** The carbimazole sample was purchased at TCI (Tokyo, Japan) (CAS RN 22232-54-8). 

**Differential Scanning Calorimetry (DSC).** DSC was performed by heating at 10 K/min under dry nitrogen atmosphere using a Perkin Elmer (Waltham, MA, USA) DSC8500 calorimeter.

**Thermo Gravimetric Analysis (TGA)**. TGA measurements were performed with a thermogravimetric analyzer TGA4000 (Perkin Elmer) in a temperature range 20 °C to 600 °C, with a rate of 10 °C/min under dry nitrogen atmosphere (flow rate 20 mL/min).

**Powder X-ray diffraction (PXRD)**. The PXRD spectrum was collected on a powdered sample using a Bruker (Rheinstetten, Germany) D8 Advance diffractometer with CuKα radiation (λ = 1.54 Å) and a LynxEye detector, operating in Bragg–Brentano geometry. Scans were recorded at room temperature (300 K) in angles ranging from 6 to 60 (°2Theta), with a step size of 0.03, and continuous scan mode.

**NMR Methods.** Solid State NMR spectra were recorded on a Bruker Avance Neo spectrometer working at Larmor frequencies of 500.13 and 125.77 MHz for ^1^H and ^13^C nuclei, respectively, equipped with triple-resonance CP-MAS probehead accommodating rotors, with an external diameter of 2.5 mm. The 90 degree pulse duration was 2.08 and 5 μs for ^1^H and ^13^C nuclei, respectively. The ^1^H-^13^C CP-MAS spectrum was recorded at a MAS frequency of 22 kHz, using a contact time of 2 ms and accumulating 1000 scans. The ^1^H MAS spectrum was recorded at a MAS frequency of 22 kHz accumulating 4 scans. The ^1^H PMLG-MAS spectrum [54] was recorded at a MAS frequency of 15 kHz accumulating 32 scans. The ^1^H DUMBO-MAS spectrum [55] was recorded at a MAS frequency of 12 kHz accumulating 32 scans. The ^1^H-^13^C HETCOR spectrum with FSLG decoupling in the indirect dimension [56] was recorded at a MAS frequency of 15 kHz, using a contact time of 0.5 ms, accumulating 128 rows and 64 scans. The ^1^H-^1^H DQ-SQ spectrum [57] was recorded at a MAS frequency of 12 kHz, using the eDUMBO-1_22_ scheme [58] for decoupling during acquisition, accumulating 256 rows and 16 scans. In all relevant experiments, a SPINAL-64 decoupling scheme [59] was applied on ^1^H nuclei while acquiring the ^13^C signal. In all cases, the measurements were carried out at room temperature (about 296 K) and a recycle delay of 10 s was used.

**Computational simulations.** All DFT calculations were performed by using the Quantum Espresso (QE) suite of programs [60], employing plane-augmented-wave (PAW) pseudopotentials [61], the PBE-D2 XC-functional [62,63], and plane-waves as basis sets to build Bloch states, with proper periodic boundary conditions inside the unit cell, determined by the experimental data of the JOVDIH01 structure (crystal axes measuring 7.698 Å, 6.650 Å, and 17.388 Å, within an orthorhombic cell belonging to the Pnma space group) [43]. Cut-offs on the wave function and electronic density were set to 60/600 Ry (1 Ry = 313.8 Kcal/mol) and the first Brillouin cell in the reciprocal space was sampled according to a (4 × 4 × 2) mesh of k points. Calculations were performed spin-restricted by applying a Gaussian smearing of the one-particle energy levels of 0.002 Ry. NMR chemical shifts (CS) were simulated by using the GIPAW approach [19] implemented in QE. For comparison with the experimental NMR data, the absolute isotropic shielding values (σ, ppm) obtained by DFT were transformed into isotropic chemical shifts (δ, ppm) through a linear least-squares fitting, which, for the calculations reported in Table 2, gave the results reported in Table 5.

## 4. Conclusions

In this work, the crystalline structure of carbimazole was deeply investigated by a combined solid state NMR-DFT approach, also exploiting previously reported XRD data. The carbimazole sample was subjected to a preliminary screening (by DSC, TGA, and PXRD), which confirmed the equivalence between its crystalline form and the form previously described in the literature and characterized by XRD [42,43]. Afterwards, isotropic ^1^H and ^13^C chemical shifts were experimentally determined by high-resolution solid state NMR techniques, offering the best accuracy, e.g., using DUMBO-MAS to obtain a well resolved ^1^H spectrum. The experimental isotropic chemical shifts were quantitatively compared with those calculated by DFT-GIPAW methods for different structures (those reported in the literature as barely derived from XRD data, and those obtained after DFT optimization of the positions of H atoms only, or of the whole molecule). The agreement achieved for the optimized structure was excellent, the RMSD values obtained, reflecting the state of the art in NMR crystallography (about 1% of the whole spectral range explored by each nucleus, i.e., 0.2 and 2 ppm for ^1^H and ^13^C, respectively [48]). The refined structure differs from the XRD structure almost exclusively for the position of H atoms, which could be determined very accurately. Importantly, the refined structure was further deeply validated through the analysis of two 2D-correlation experiments (^1^H-^13^C HETCOR and ^1^H-^1^H DQSQ), whose signals intensities were all found to be in excellent (although semi-quantitative) agreement with the strengths of the dipolar couplings predicted from the inter-nuclear distances of the optimized structures. Here, the relevant role of inter-molecular dipolar interactions for specific chemical groups (e.g., methyl groups 4 and 7) was clearly observed and highlighted. Deviations from a fully quantitative agreement between 2D signal intensities and calculated dipolar coupling strengths must be at least partially ascribed to vibrational and inter-conformational motions, which can be effectively investigated by solid-state NMR through the measurement of interaction anisotropies and relaxation times [64,65,66,67]. This will be the subject of a future paper.

This study clearly confirms the importance and reliability of NMR crystallography, especially in the pharmaceutical field. To the best of our knowledge, this is the first time that solid-state NMR experiments were performed on carbimazole.

## Figures and Tables

**Table 1:** Comparison of the two crystal structures of carbimazole JOVDIH [42] and JOVDIH01 [43] reported in the literature.

	JOVDIH		JOVDIH01	
Space Group	P n a 2_1_		P n m a	
Unit Cell	a = 7.689(2)Å b = 17.364(4)Å c = 6.637(1)Å	α = 90° β = 90° γ = 90°	a = 7.698(3)Å b = 6.650(3)Å c = 17.388(7)Å	α = 90° β = 90° γ = 90°
Z Z′	4 1		4 1	
R factor	0.06		-	
Radiation	CuKα $\bar{\lambda }=1.541\;78$ Å		-	
Temperature	Room Temperature		-	

**Table 2:** Experimental and calculated isotropic chemical shifts (δ) of ^1^H and ^13^C nuclei and the corresponding assignment. Three different calculated values are reported, obtained from: (i) X-ray structure [42,43], (ii) structure obtained optimizing the positions of H atoms only, and (iii) structure obtained optimizing the positions of all atoms. Differences between calculated and experimental δ values are reported in parentheses soon after the calculated values. Root mean square deviations (RMSD) between experimental and calculated values are reported for the three levels of calculations.

Assignment	Experimental Chemical Shift δ (ppm)	δ at the DFT Level X-ray Structure (ppm) JOVDIH01	δ at the DFT Level X-ray Structure (ppm) JOVDIH	δ at the DFT Level Only H Optimized (ppm) JOVDIH01	δ at the DFT Level Only H Optimized (ppm) JOVDIH	δ at the DFT Level All Atoms Optimized (ppm) JOVDIH01	δ at the DFT Level All Atoms Optimized (ppm) JOVDIH
H7	2.31	2.28 (−0.03)	1.96 (−0.35)	2.36 (+0.05)	2.33 (+0.02)	2.46 (+0.15)	2.44 (+0.13)
H4	4.28	4.20 (−0.08)	4.68 (+0.40)	4.30 (+0.02)	4.26 (−0.02)	4.16 (−0.12)	4.19 (−0.09)
H6	5.59	5.77 (+0.18)	5.85 (+0.26)	5.45 (−0.14)	5.56 (−0.03)	5.44 (−0.15)	5.42 (−0.17)
H2	6.85	7.17 (+0.32)	6.78 (−0.07)	6.84 (−0.01)	6.87 (+0.02)	6.80 (−0.05)	6.79 (−0.06)
H3	7.85	7.58 (−0.27)	7.61 (−0.24)	7.92 (+0.07)	7.86 (+0.01)	8.02 (+0.17)	8.03 (+0.18)
RMSD	-	0.19	0.29	0.07	0.02	0.13	0.13
C7	16.0	10.9 (−5.1)	11.2 (−4.8)	13.6 (−2.4)	13.2 (−2.8)	14.1 (−1.9)	14.1 (−1.9)
C4	35.6	36.5 (+0.9)	37.3 (+1.7)	35.7 (+0.1)	35.8 (+0.2)	34.9 (−0.7)	35.1 (−0.5)
C6	66.8	68.6 (+1.8)	70.6 (+3.8)	68.8 (+2.0)	71.1 (+4.3)	69.2 (+2.4)	68.9 (+2.1)
C2	111.0	116.5 (+5.5)	110.7 (−0.3)	111.6 (+0.6)	108.4 (−2.6)	110.7 (−0.3)	110.8 (−0.2)
C3	119.8	123.7 (+3.9)	123.0 (+3.2)	121.9 (+2.1)	122.1 (+2.3)	122.1 (+2.3)	122.2 (+2.4)
C5	148.7	147.8 (−0.9)	148.3 (−0.4)	149.1 (+0.4)	148.6 (−0.1)	151.1 (+2.4)	151.1 (+2.4)
C1	166.0	159.8 (−6.2)	162.7 (−3.3)	163.0 (−3.0)	164.5 (−1.5)	161.7 (−4.3)	161.6 (−4.4)
RMSD	-	4.0	3.0	1.8	2.4	2.4	2.4

**Table 3:** Signals in the ^1^H-^13^C HETCOR spectrum, numbered in order of decreasing intensity. For each signal, the nuclei involved in the interaction, the normalized intensity, and the minimum distance between the nuclei calculated from the optimized crystal structure are reported. Asterisks in the coupled nuclei column denote intermolecular interactions.

Peak No.	Coupled Nuclei	Intensity	Distance (Å)
1	C7-H7	1.00	1.1
2	C4-H4	0.99	1.1
3	C6-H6	0.41	1.1
4	C2-H2	0.30	1.1
5	C3-H3	0.29	1.1
6	C6-H7	0.20	2.1
7	C4-H7 *	0.19	3.4
8	C7-H6	0.19	2.2
9	C7-H4 *	0.12	3.2
10	C4-H3	0.12	2.8
11	C3-H4	0.08	2.6
12	C2-H7 *	0.07	3.6
13	C3-H6 *	0.07	3.3
14	C2-H4 *	0.06	3.4
15	C3-H7 *	0.05	4.6

* Intermolecular interactions.

**Table 4:** Signals of the ^1^H-^1^H DQSQ spectrum, numbered and reported in order of decreasing intensity. For each signal, the pair of protons involved in the interaction, the normalized intensity, and the minimum distance between the nuclei calculated from the optimized crystal structure are reported.

Peak No.	Coupled Nuclei	Intensity	Distance (Å)
1	H4-H4	1.00	1.8
2	H6-H6	0.63	1.8
3	H7-H7	0.50	1.8
4	H4-H7 *	0.33	2.5
5	H3-H6 *	0.28	2.5
6	H2-H7 *	0.27	2.5
7	H4-H3	0.23	2.4
8	H2-H4 *	0.22	2.8
9	H4-H2 *	0.21	2.8
10	H6-H3 *	0.20	2.5
11	H7-H6	0.20	2.5
12	H7-H2 *	0.20	2.5
13	H6-H7	0.19	2.5
14	H7-H4 *	0.19	2.5
15	H3-H4	0.17	2.4
16	H3-H2	0.17	2.8
17	H2-H3	0.17	2.8

* Intermolecular interactions.

**Table 5:** Relationships between ^13^C and ^1^H absolute isotropic shielding values (σ, ppm) calculated by DFT and corresponding isotropic chemical shifts (δ, ppm), as obtained through a linear least-squares fitting to the experimental δ values.

	^13^C	R^2^	^1^H	R^2^
X-ray structure JOVDIH01	δ = −1.2011 σ + 202.54	0.994	δ = −1.3808 σ + 37.025	0.990
X-ray structure JOVDIH	δ = −1.0713 σ + 180.07	0.997	δ = −1.0600 σ + 32.128	0.979
only H optimized JOVDIH01	δ = −0.9883 σ + 172.46	0.999	δ = −0.9975 σ + 31.289	0.999
only H optimized JOVDIH	δ = −1.0020 σ + 170.7	0.998	δ = −1.0114 σ + 31.299	1.000
all atoms optimized JOVDIH01	δ = −1.0259 σ + 172.13	0.998	δ = −1.0596 σ + 31.302	0.995
all atoms optimized JOVDIH	δ = −1.0253 σ + 172.22	0.998	δ = −1.0628 σ + 31.481	0.995

## Data Availability

The data presented in this study are available on request from the corresponding author.

## Supplementary Materials

The following are available online, Figure S1: Simulated XRPD spectra of carbimazole, Table S1: RMSD of the atom positions of the optimized structure with respect to the SCXR structure.

## Sample Availability

Samples of the compounds are not available from the authors.
