# Supplementary material for: Structural Refinement of Carbimazole by NMR Crystallography
Source: Molecules. 2021 Jul 29;26(15):4577. doi: 10.3390/molecules26154577 (PMC8347463; doi:10.3390/molecules26154577)
Supplement: Supplementary file 1 [file molecules-26-04577-s001.zip › molecules-1301376-supplementary.pdf]

Supplementary Materials

Structural refinement of carbimazole by NMR crystallography

Andrea Scarperi, Giovanni Barcaro, Aleksandra Pajzderska, Francesca Martini, Elisa Carignani*, Marco Geppi*

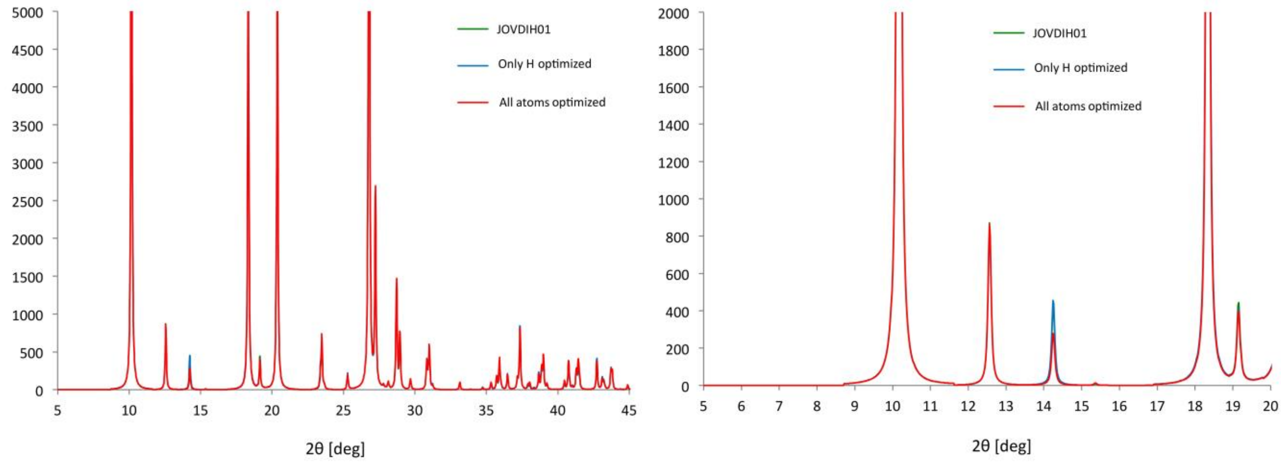

Figure S1. Simulated XRPD spectra from JOVIH01 [1] (green) and two levels of structural optimization: only H (blue) and full optimization (red).

Table S1. Root mean square deviations (RMSD) of atoms positions of the optimized structures with respect to the SCXR crystal structures of carbimazole JOVDIH01 [1] and JOVDIH [2]. A careful analysis did not highlight any particular hydrogen atom changing more than the others after optimization.

Atom type (quantity in minimal unit)	RMSD DFT-Exp Only H optimized (Angstrom) JOVDIH01	RMSD DFT-Exp Only H optimized (Angstrom) JOVDIH	RMSD DFT-Exp All atoms optimized (Angstrom) JOVDIH01	RMSD DFT-Exp All atoms optimized (Angstrom) JOVDIH
**C (7)**	0.00	0.00	0.04	0.06
**N (2)**	0.00	0.00	0.03	0.05
**S (1)**	0.00	0.00	0.02	0.00
**O (2)**	0.00	0.00	0.04	0.04
**H (10)**	0.09	0.05	0.14	0.13

References

1. Das, D.; Roy, G.; Mugesh, G. Antithyroid Drug Carbimazole and Its Analogues: Synthesis and Inhibition of Peroxidase-Catalyzed Iodination of L-Tyrosine. *J. Med. Chem.* **2008**, *51*, 7313–7317, doi:10.1021/jm800894m.
2. Delage, C.; Faure, F.; Leger, J.M.; Raby, C.; Goursolle, M. Conformational study of 3-methyl 2-thio imidazoline ethyl 1-carboxylate. *C. R. Acad. Sci. Paris* **1990**, *311*, 781–784.
